# Supplementary material for: Causal Relationship between Aging and Anorexia Nervosa: A White-Matter-Microstructure-Mediated Mendelian Randomization Analysis
Source: Biomedicines. 2024 Aug 16;12(8):1874. doi: 10.3390/biomedicines12081874 (PMC11351342; doi:10.3390/biomedicines12081874)
Supplement: Supplementary file 1 [file biomedicines-12-01874-s001.zip › Supplementary Figure/Figure S9/Leaveoneout plot of MR analysis for ubm-b-1627 to AN.pdf]

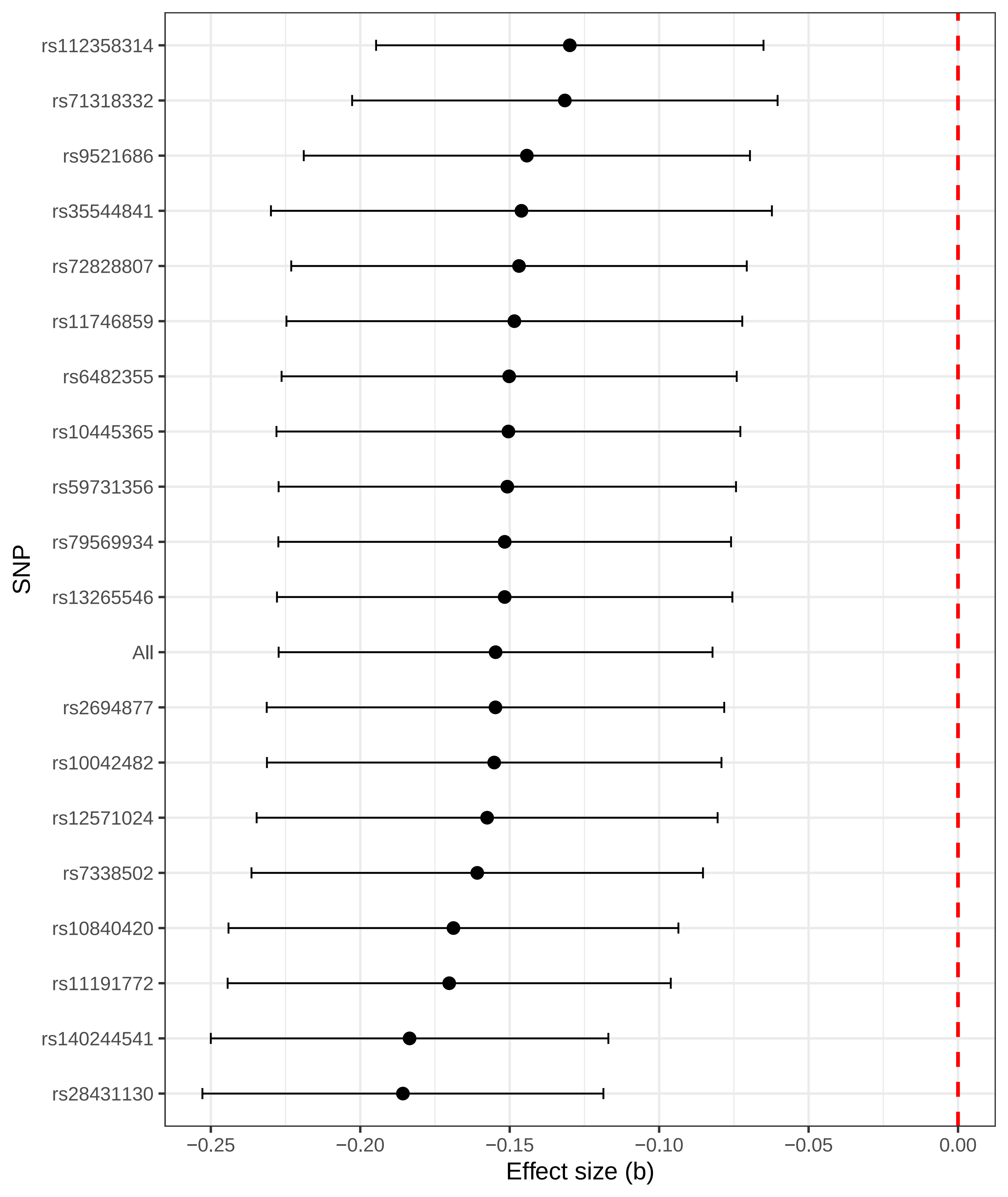

rs112358314
rs71318332
rs9521686
rs35544841
rs72828807
rs11746859
rs6482355
rs10445365
rs59731356
rs79569934
rs13265546
All
rs2694877
rs10042482
rs12571024
rs7338502
rs10840420
rs11191772
rs140244541
rs28431130
SNP
−0.25
−0.20
−0.15
−0.10
−0.05
0.00
Effect size (b)
